# Knock-down of NDRG2 sensitizes cervical cancer Hela cells to cisplatin through suppressing Bcl-2 expression

**DOI:** 10.1186/1471-2407-12-370

**Published:** 2012-08-27

**Authors:** Junye Liu, Le Yang, Jian Zhang, Jing Zhang, Yongbin Chen, Kangchu Li, Yurong Li, Yan Li, Libo Yao, Guozhen Guo

**Affiliations:** 1 Department of Radiation Medicine, Fourth Military Medical University, Xi’an, China; 2 Department of Biochemistry and Molecular Biology, State Key Laboratory of Cancer Biology, Fourth Military Medical University, Xi’an, China; 3 Department of Oncology, Xi’an Central Hospital, Xi’an, China

**Keywords:** Bcl-2, Chemosensitivity, Cisplatin, NDRG2, RNA interference

## Abstract

**Background:**

NDRG2, a member of N-Myc downstream regulated gene family, plays some roles in cellular stress, cell differentiation and tumor suppression. We have found that NDRG2 expression in cervical cancer Hela cells increases significantly upon stimulation with cisplatin, the most popular chemotherapeutic agent currently used for the treatment of advanced cervical cancer. This interesting phenomenon drove us to evaluate the role of NDRG2 in chemosensitivity of Hela cells.

**Methods:**

In the present study, RNA interference was employed to down-regulate NDRG2 expression in Hela cells. RT-PCR and Western blot were used to detect expression of NDRG2, Bcl-2 and Bax in cancer cells. Real-time PCR was applied to detect miR-15b and miR-16 expression levels. Drug sensitivity was determined with MTT assay. Cell cloning efficiency was evaluated by Colony-forming assay. Apoptotic cells were detected with annexin V staining and flow cytometry.

**Results:**

In vitro drug sensitivity assay revealed that suppression of NDRG2 could sensitize Hela cells to cisplatin. Down-regulation of NDRG2 didn’t influence the colony-forming ability but promoted cisplatin-induced apoptosis of Hela cells. Inhibition of NDRG2 in Hela cells was accompanied by decreased Bcl-2 protein level. However, Bcl-2 mRNA level was not changed in Hela cells with down-regulation of NDRG2. Further study indicated that miR-15b and miR-16, two microRNAs targetting Bcl-2, were significantly up-regulated in NDRG2-suppressed Hela cells.

**Conclusions:**

These data suggested that down-regulation of NDRG2 could enhance sensitivity of Hela cells to cisplatin through inhibiting Bcl-2 protein expression, which might be mediated by up-regulating miR-15b and miR-16.

## Background

Cervical cancer is the second largest cause of cancer mortality in women worldwide with more than 270,000 deaths per year [1]. Current therapies for the treatment of advanced cervical cancer involve the use of cisplatin, often in combination with radiotherapy [2]. Cisplatin is believed to act via the formation of inter- and intrastrand cross-links in DNA, culminating in the initiation of cell death via caspases [3]. Unfortunately, the current cisplatin-based treatment for cervical cancer does not lead to a high disease-free survival rate in patients with bulky or locally-advanced disease. To develop new potentially therapeutic treatments for cancers, two strategies have been developed. The first strategy uses an approach to identify potential mechanisms of resistance to cancer therapy, and to overcome these resistance phenotypes using specific resistance modulators [4]. The second strategy seeks to correlate biological features with genetic alterations in cancer cell [5]. Although some progress has been achieved in the past three decades, much more efforts are still needed to resolve cisplatin-resistance of cervical cancer.

NDRG2, a member of N-Myc downstream regulated gene (NDRG) family, was first cloned in our laboratory in 1999 (GenBank accession no. AF159092). NDRG gene family represents a new class of Myc-repressed genes which also consist of NDRG1, NDRG3 and NDRG4 [6]. The NDRGs share 57-65% amino acid identity and are highly conserved in plants, invertebrates and mammals, suggesting that this gene family may have important cellular functions. Although NDRG2 has been implicated in cellular stress [6], it’s physiological function still remains unclear. Interestingly, NDRG2 has been found to be deregulated in many kinds of human malignant tumors [7-17]. We previously reported that NDRG2 could be up-regulated by hypoxia and radiation and could promote radioresistance of human cervical cancer Hela cells [18]. In another study, we found adriamycin enhanced NDRG2 expression in several tumor cell lines [19]. This led us to further explore whether NDRG2 has a role in regulation of cisplatin-sensitivity of cervical cancer cells.

## Methods

### Cell culture

The human cervical cancer cell line Hela was obtained from the American Type Culture Collection (Manassas, VA) and maintained as a monolayer in Dulbecco’s modified Eagle’s medium (Invitrogen, Carlsbad, CA) supplemented with 10% fetal bovine serum (Sijiqing Biological Engineering Materials Co., Hangzhou, China) at 37°C in the presence of 5% CO_2_-balanced air.

### Constructs and transfection

The recombinant pSilencer 3.1 (Ambion, Austin, TX) constructs expressing a scramble control small interference RNA (siRNA) or siRNA specific to NDRG2 have been described previously [20]. All construct sequences were directly confirmed by DNA sequencing. Hela cells were transfected with the corresponding constructs using Lipofectamine^TM^ 2000 (Invitrogen, Carlsbad, CA) according to the manufacturer’s instruction.

### RT-PCR

Total RNA was extracted from Hela cells using Trizol reagent according to instructions provided by the manufacturer (Invitrogen, Carlsbad, CA). cDNAs were obtained by reverse transcription using Revert Aid First Strand cDNA Synthesis Kit (Fermentas, Shenzhen, Guangzhou, China) with 2 μg of total RNA. The RT-PCR exponential phase was determined on 25–30 cycles to allow semi-quantitative comparisons among cDNAs developed from identical reactions. Each PCR involved a 94°C, 5 min initial denaturation step followed by 35 cycles (for NDRG2) at 94°C for 30 s, 52°C for 40 s, and 72°C for 40 s, 20 cycles (for β-actin) at 94°C for 30 s, 55°C for 10 s, and 72°C for 30 s, 25 cycles (for Bcl-2) at 94°C for 30 s, 62°C for 30 s, and 72°C for 30 s, then 72°C, 10 min extension step. Oligonucleotide primer sequences for NDRG2 [20], Bcl-2 [21] and β-actin [21] have been described previously. The PCR products were separated by electrophoresis on 1.5% agarose gels.

### Real-time PCR

NDRG2 and Bcl-2 mRNA expression levels were evaluated by real-time PCR using the ABI PRISM 5700 sequence detection system (Applied Biosystems, Foster City, CA). Primers for NDRG2 have been described previously [12]. The primer sequences for Bcl-2 were: 5'-GGTGAACTGGGGGAGGATTGT-3' for the forward primer, and 5'-CTTCAGAGACAGCCAGGAGAA-3' for the reverse primer. Fluorescent data were converted into cycle threshold measurements using the SDS system software and exported to Microsoft Excel. Fold expression changes relative to Hela cells were calculated with the ΔΔCT method [22] using ARF1 (ADP-ribosylation factor 1) as the reference transcript [12].

For detection of microRNAs (miRNA), stem-loop reverse transcription followed by real-time PCR analysis was performed as previously described [21]. The primers used for stem-loop RT-PCR for Let-7a [23], miR-15b [21] and miR-16 [21] have been reported elsewhere. The relative amount of each miRNA was normalized to U6 snRNA. The fold-change for each miRNA relative to the Hela cells was calculated using ΔΔCT method [22]. PCR was performed in triplicate.

### Western blot analysis

Hela cells and their variants were solubilized in lysis buffer containing 20 mM Tris, pH 7.4, 150 mM NaCl, 1% Triton X-100, 5 mM EDTA, 10 μg/ml leupeptin, 10 μg/ml aprotinin, 1 mM phenylmethylsulfonyl fluoride. Fifty micrograms of cell lysate was resolved by SDS-PAGE and transferred to nitrocellulose membranes (0.22 μm, Millipore, Bedford, MA). The blots were probed with antibodies (from Santa Cruz Biotechnology, Santa Cruz, CA, unless otherwise indicated) against NDRG2, NDRG1, Bcl-2, Bax, P-glycoprotein, multidrug resistance protein, caspase-3 (Cell Signaling Technology, Danvers, MA) or caspase-9 (Cell Signaling Technology), followed by incubation in a species-matched horseradish peroxidase (HRP)-conjugated secondary antibody. The blots were developed with a chemiluminescence substrate solution (Pierce, Rockford, IL) and exposed to X-ray film. Equal loading of all lanes was confirmed by reprobing the membranes with anti-β-actin antibody (Sigma, St. Louis, MO).

### Cytotoxicity assay

The effect of cisplatin on growth of Hela and its variants was determined by 3-(4,5-Dimethylthiazol-2-yl)-2,5-diphenyl-tetrazolium bromide (MTT) assay as previously described [24]. Briefly, 2000 cells were plated into 96-well plates in 200 μl aliquots and allowed to adhere overnight before cisplatin (Qilu Pharmaceutical Co Ltd, Jinan, Shandong, China) was added. The final concentrations of cisplatin were as 0.01, 0.1, 1, 10 and 100 times of human peak plasma concentration of cisplatin (2 μg/ml) [24], respectively. After incubation with cisplatin at 37°C for 72 h, 20 μl aliquots of an MTT solution (5 mg/ml) were added to each well and plates were incubated for an additional 4 h at 37°C. The media was then removed and replaced with 150 μl of 100% DMSO to dissolve the formazan crystals with agitation for 5–10 min on a shaker. The absorbance was measured at 490 nm using a multiwell scanning spectrophotometer (Molecular Devices, Sunnyvale, CA). Cell viability was expressed as A490 percentage of untreated cells. The IC_50_ values were determined from a plot of cell viability using probit module of SPSS software (version 10.0; SPSS, Chicago, IL). Three replicates were performed for each experimental condition.

### Colony-forming assay

Cells were seeded on six-well tissue culture plates with 500 cells per well and incubated in complete medium with or without cisplatin (2, 5, 10 μg/ml). After 12 days of culture, colonies of surviving cells were counted and the cloning efficiency was calculated in comparison to seeded cells.

### Detection of apoptosis

Hela cells and their variants were treated with cisplatin at desired concentration for 24 h. The apoptotic cells were stained with fluorescein isothio-cyanate (FITC)-labeled annexin V (Roche Applied Science, Basel, Switzerland) and propidium iodide (PI) as previously described [18]. Apoptotic cells were measured using a Becton Dickinson fluorescence-activated cell sorter (FACS) apparatus.

### Statistical analysis

Data are expressed as mean ± SD. Statistical analyses were performed with the SPSS software (version 10.0; SPSS, Chicago, IL) by using one-way ANOVA followed by the *t*-test for independent groups. A p level of < 0.05 was considered statistically significant.

## Results

### NDRG2 expression was enhanced in Hela cells exposed to cisplatin

**Figure 1:**
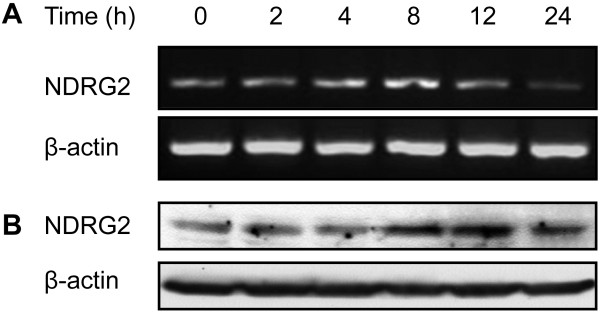
**NDRG2 expression in Hela cells was enhanced by cisplatin.** Hela cells were exposed to cisplatin (5 μg/ml) for the indicated time. NDRG2 expression was evaluated by RT-PCR (**A**) and Western blot (**B**) analysis. β-actin served as internal control. Shown are representative of 3 independent experiments.

In the present study, the effect of cisplatin on NDRG2 expression was evaluated in human cervical cancer Hela cells. Hela cells were exposed to cisplatin at concentration of 2, 5, 7,10 μg/ml, respectively. It was found cisplatin up-regulated NDRG2 expression in Hela cells, with the most prominent effects of cisplatin on NDRG2 expression were seen at concentration of 5 μg/ml (data not shown). As indicated in Figure 1, NDRG2 mRNA (Figure 1A) and protein (Figure 1B) levels significantly increased upon incubation with 5 μg/ml of cisplatin. The change of NDRG2 was time-dependent and sustained at least for 24 h. These suggested that NDRG2 might be involved in drug response and chemosensitivity of Hela cells.

### Down-regulation of NDRG2 sensitized Hela cells to cisplatin

**Figure 2:**
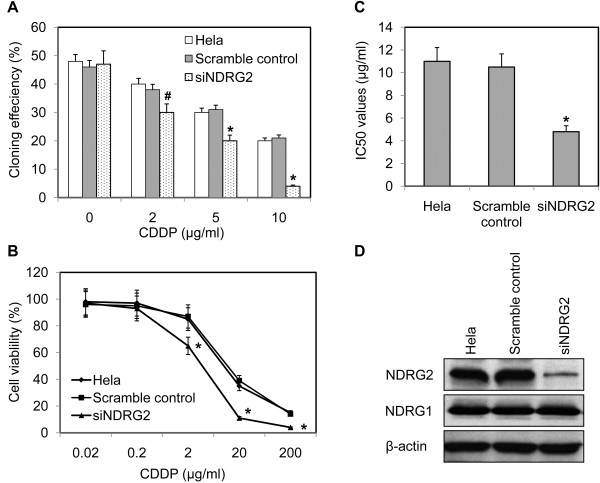
**Down-regulation of NDRG2 sensitized Hela cells to cisplatin.** Hela cells were transfected with pSilencer3.1 constructs expressing NDRG2-specific siRNA (siNDRG2) or scramble control. The G418-resistant mix clones were selected for further assays. **A**. The colony-formation ability of Hela cells and their variants was evaluated in the presence or absence of cisplatin (CDDP). **B**. Cell viability was evaluated by MTT assay as described in “Materials and Methods” and plotted against CDDP concentration. **C**. The IC_50_ values were determined according to the plot. * p < 0.01 vs Hela and scramble control. **D**. Down-regulation of NDRG2 in stable clones was displayed by Western blot with β-actin as loading control. Shown are representative of 3 independent experiments. * p < 0.01 vs Hela and scramble control. # p < 0.05 vs Hela and scramble control.

To address the role of NDRG2 in chemosensitivity of Hela cells, Hela cells were transfected with DNA constructs expressing NDRG2-specific siRNA or scramble control. The G418-resistant mix clones were selected for further experiments. As shown in Figure 2D, NDRG2-specific siRNA resulted in significantly inhibited NDRG2 expression with no obvious change of NDRG1 in Hela cells. Colony-forming assay was employed to determine the effect of NDRG2 on cell growth. It was found that inhibition of NDRG2 didn’t alter the colony-forming ability in the absence of cisplatin but did enhance the suppressive effect of cisplatin on colony-forming ability of Hela cells (Figure 2A). The cell viability in the presence of cisplatin was further evaluated with MTT assay. It was indicated down-regulation of NDRG2 resulted in poor cell viability (Figure 2B). The IC_50_ values of Hela cells to cisplatin were calculated from the cell viability plots. As displayed by Figure 2C, suppression of NDRG2 was accompanied by significantly decreased IC_50_ values. These data indicated that down-regulation of NDRG2 could increase sensitivity of Hela cells to cisplatin.

### Down-regulation of NDRG2 enhanced cisplatin-induced apoptosis

**Figure 3:**
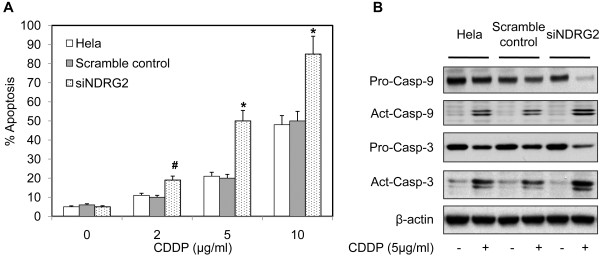
**Down-regulation of NDRG2 enhanced cisplatin-induced apoptosis of Hela cells.** Hela cells and the stable variants were exposed to different concentration of cisplatin (CDDP) for 24 h. The apoptotic cells were either stained with annexin V-FITC and propidium iodide (PI) and examined with flow cytometry, or subjected to Western blot. **A**. The percentage of apoptotic cells was presented. # p < 0.05 vs Hela and scramble control. * p < 0.01 vs Hela and scramble control. **B**. The expression of caspase-3 (Casp-3) and caspase-9 (Casp-9) was detected by Western blot analysis using β-actin as loading control. Shown are representative of 3 independent experiments. Act, active caspase. Pro, procaspase.

The mechanisms underlying NDRG2 effect on chemosensitivity were further explored. Considering NDRG2 has no influence on colony-forming ability and most kinds of chemotherapeutic drugs inhibit tumor growth through inducing apoptosis of cancer cells, the effect of NDRG2 on cisplatin-induced apoptosis was evaluated. Hela cells and their stable variants were treated with cisplatin at different concentration and apoptotic cells were examined with flow cytometry. In the absence of cisplatin, Hela cells and their variants displayed similar spontaneous apoptotic rate (Figure 3A). Treatment of cisplatin increased apoptosis of each kind of cells at dose-dependent manner. Hela cells with down-regulated NDRG2 had highest apoptotic rate among those 3 kinds of cells (Figure 3A), indicating inhibition of NDRG2 significantly enhanced cisplatin-induced apoptosis of Hela cells.

To validate the effect of NDRG2 on apoptosis, we further analyzed the proteolytic maturation of caspase-9 and caspase-3 triggered by CDDP. As indicated in Figure 3B, whereas CDDP induced a minor yet detectable increase in the abundance of the cleaved fragments of both caspase-9 and caspase-3 in Hela and scramble control cells, siNDRG2 cells responded to CDDP by massively activating caspases as well as significantly decreased procaspase-9 and procaspase-3. Comparing to scramble control, siNDRG2 cells exhibited similar levels of procaspase-9 and procaspase-3 (Figure 3B), suggesting no obvious influence of NDRG2 on caspase expression.

### Suppression of NDRG2 inhibited Bcl-2 expression in Hela cells

**Figure 4:**
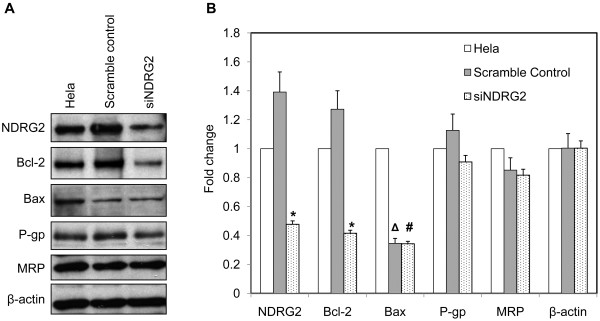
**Suppression of NDRG2 inhibited Bcl-2 expression in Hela cells. A**. The expression of Bcl-2, Bax, P-glycoprotein (P-gp) and multidrug resistance protein (MRP) in Hela and its stable variants was evaluated by Western blot with β-actin as loading control. Shown are representative of 3 independent experiments. **B**. Densitometric analysis of Western blot assay was further performed. Data was expressed as fold change using Hela cells as reference. * p < 0.01 vs Hela and scramble control. Δ p < 0.01 vs Hela. # p > 0.05 vs scramble control.

To further illustrate the role of NDRG2 in cisplatin-induced apoptosis, the expression of Bax and Bcl-2 was examined in Hela cells and their variants by Western blot. Comparing to Hela cells, the scramble control cells showed slightly but not significantly increased levels of NDRG2 and Bcl-2 (Figure 4). siNDRG2 cells exhibited down-regulation of NDRG2 as well as significantly repression of Bcl-2 in comparison with Hela and scramble control (Figure 4). Although both scramble control and siNDRG2 cells showed much lower level of Bax than Hela cells, there was no significant difference of Bax between scramble control and siNDRG2 cells (Figure 4), indicating no obvious relation of Bax to knock-down of NDRG2. In addition, P-glycoprotein and multidrug resistance protein, two transporters associated with cancer drug resistance including cisplatin resistance [25], were not influenced by NDRG2 in Hela cells (Figure 4). These data suggested that down-regulation of NDRG2 specifically inhibited Bcl-2 expression and reduced the Bcl-2/Bax ratio, which might in turn increase the sensitivity of Hela cells to cisplatin-induced apoptosis.

### NDRG2 modulated Bcl-2 expression through regulating miR-15b and miR-16

**Figure 5:**
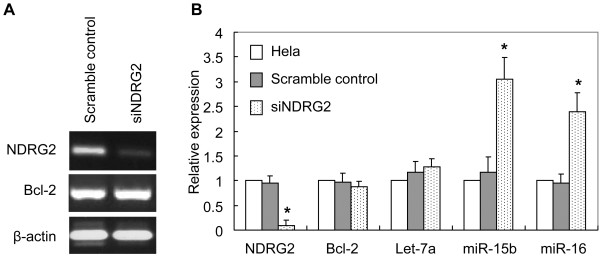
**Suppression of NDRG2 did not change Bcl-2 mRNA level but increased expression of miR-15b and miR-16 in Hela cells.** The mRNA expression of NDRG2 and Bcl-2 was evaluated by RT-PCR (**A**) with β-actin as loading control. The expression of NDRG2, Bcl-2 and microRNAs (let-7a, miR-15b, miR-16) in Hela and its stable variants was determined by real-time PCR (**B**) as described in “Materials and Methods”. Fold expression changes relative to Hela cells were calculated. * p < 0.01 vs Hela and scramble control.

The mRNA level of Bcl-2 in Hela cells and their variants was determined to address if NDRG2 transcriptionally regulated Bcl-2 expression. As revealed by RT-PCR, Hela cells expressing NDRG2-specific siRNA exhibited reduced NDRG2 mRNA level in comparison with scramble control cells (Figure 5A). However, Bcl-2 mRNA level was not changed by down-regulation of NDRG2 (Figure 5A). Real-time PCR was further employed to detect Bcl-2 and NDRG2 expression in Hela cells and their variants. It was shown Hela cells and their variants harbored similar level of Bcl-2 mRNA (Figure 5B), indicating NDRG2 regulated Bcl-2 expression at post-transcriptional level. miR-15b and miR-16, two microRNAs that had been reported to target Bcl-2 and inhibit Bcl-2 protein expression [21], were evaluated in Hela cells and their variants. It was found miR-15b and miR-16 significantly increased in Hela cells expressing NDRG2-specific siRNA comparing to Hela parental cells and scramble control (Figure 5B). Another microRNA, Let-7a, was not changed in those 3 kinds of cells. These data suggested that down-regulation of NDRG2 resulted in increased expression of miR-15b and miR-16, which subsequently inhibited translation of Bcl-2 and led to reduced expression of Bcl-2 protein.

## Discussion

Platinum-based drugs, and in particular cisplatin, are widely used for the treatment of many kinds of solid malignancies, including cervical cancer [2]. Cisplatin often leads to an initial therapeutic success associated with partial responses or disease stabilization. However, some patients developed resistance to cisplatin before and even after exposing to this chemotherapeutic agent. It is widely accepted that the high incidence of chemoresistance is the main reason for cisplatin treatment failure. Although the pre-target, on-target, post-target and off-target mechanisms of cisplatin-resistance have been proposed recently [25], the molecular mechanisms underlying cisplatin-resistance are still far from understood.

In the present study, we provide evidence that NDRG2 is involved in the regulation of cisplatin-resistance of cervical cancer cells. Firstly, it was shown that cisplatin treatment induced up-regulation of NDRG2 in a time-dependent manner. Secondly, knock-down of NDRG2 by siRNA increased the suppressive effects of cisplatin on colony-forming ability of Hela cells. Thirdly, inhibition of NDRG2 significantly lowered the IC_50_ values of cisplatin for Hela cells, indicating down-regulation of NDRG2 increased the sensitivity of Hela cells to cisplatin. Finally, down-regulation of NDRG2 resulted in decreased expression level of Bcl-2, one of important regulators of apoptosis and drug resistance.

NDRG2, similar to NDRG1, has been proposed to be involved in cell stress [6]. Previous studies demonstrated that NDGR2 expression in cancer cell lines could be enhanced by adriamycin [19], hypoxia [20] and radiation [18], and that NDRG2 was implicated in regulation of adriamycin-induced apoptosis [19] and radioresistance [18]. All those data support a role of NDRG2 in cell stress. It is well-characterized that the mode of action of cisplatin involves the DNA-damage response and mitochondrial apoptosis [26,27]. From this view, it is reasonable that NDRG2 can be induced by cisplatin. In line with previous reports, the present study indicates modulation of NDRG2 expression occurs at transcriptional level. Although NDRG2 transcription can be suppressed by Myc [28], several binding sites for hypoxia-inducible factor 1 (HIF-1) have been found in the promoter region of NDRG2 gene and HIF-1 is responsible for NDRG2 up-regulation induced by hypoxia and radiation [18,20]. It has been reported that HIF-1 can be activated by vincristine in gastric cancer cells under normoxic condition [29]. Cisplatin, as a chemotherapeutic drug like adriamycin and vincristine, might induce NDRG2 expression through activation of HIF-1. However, this speculation needs to be validated in future study.

It is well known that Bcl-2 and Bax play critical roles in mitochondria apoptosis. As discussed in a recently published review [25], there are increasing evidences that overexpression of Bcl-2 confers multidrug resistance and that clinical data have linked Bcl-2 expression level with cisplatin resistance and recurrent disease. The present study revealed that suppression of NDRG2 significantly inhibited Bcl-2 expression, which depictes how does NDRG2 influence cisplatin-induced apoptosis of Hela cells. Interestingly, NDRG2 regulates Bcl-2 expression at post-transcriptional level. It has been well defined that Bcl-2 can be targeted and regulated by microRNAs such as miR-15b and miR-16 in gastric cancer cells [21]. These microRNAs bind to the 3’ untranslational region of Bcl-2 mRNA and inhibit translation of Bcl-2 protein without changing mRNA level. Similarly, the present study indicated that down-regulation of NDRG2 resulted in increased level of miR-15b and miR-16, which might in turn reduce Bcl-2 protein expression. However, the study does not exclude the possibility that NDRG2 influences stabilization of Bcl-2 protein.

It has been well documented that hypoxia- and radiation-induced NDRG2 promotes resistance of Hela cells to radiation [18]. The present study demonstrated that cisplatin-induced NDRG2 increases chemoresistance of Hela cells. Considering cisplatin is often used in combination with radiotherapy for advanced cervical cancer [2], NDRG2 may represent a key regulator of therapy-resistance in cervical cancer cells. It should be noted that NDRG2 has been proposed as a potential tumor suppressor. An increasing number of reports showed that the level of NDRG2 was reduced in many kinds of malignant tumor comparing to the normal counterpart and that NDRG2 level was an independent prognostic factor for cancer patients [7-17]. It was also reported that restoration of NDRG2 could inhibit proliferation of cancer cells [6]. Recently, the crystal structure of human NDRG2 protein was resolved and NDRG2 was proposed to suppress TCF/β-catenin signaling in the tumorigenesis of human colorectal cancer via a molecular interaction [30]. Moreover, a role for NDRG2 in the oncogenic properties of renal cell carcinoma has been suggested [31]. However, these data cannot exclude the possibility of NDRG2 to promote drug and radiation resistance. We proposed NDRG2 as a multifunctional protein. As a tumor suppressor, NDRG2 is inhibited to facilitate tumor development in the process of tumorigenesis. As a resistance regulator, NDRG2 may be re-activated or up-regulated to promote cancer cell survival during or after chemotherapy/radiotherapy. In an early study, it was shown inhibition of NDRG2 resulted in slightly increased proliferation and cisplatin resistance as well as decreased Fas expression and Fas-mediated cell death in gastric cancer cells [9]. The discrepancy of NDRG2 in cisplatin resistance in cervical cancer and gastric cancer cells may due to tissue specificity. However, our speculation needs supporting data from further studies. It will be helpful to validate the role of NDRG2 in cisplatin resistance in multiple cell lines other than Hela and to explore the clinical relevance of NDRG2 to cisplatin resistance in patients with cervical cancer.

## Conclusions

The present study demonstrated that NDRG2 can be induced in Hela cells by cisplatin in a time-dependent manner and that knock-down of NDRG2 by siRNA increases sensitivity of Hela cells to cisplatin. Down-regulation of NDRG2 could enhance cisplatin-induced apoptosis of Hela cells through inhibiting Bcl-2 protein expression, which might be mediated by up-regulating miR-15b and miR-16. These data can help us to better understand the molecular mechanisms of cisplatin-resistance and the precise role of NDRG2 in tumor development.

## Abbreviations

HIF-1: hypoxia inducible factor 1; NDRG2: N-Myc downstream regulated gene 2; siRNA: small interference RNA.

## Competing interests

The authors declare that they have no competing interests.

## Authors' contributions

JL, LY, JZ, JZ, YC, KL, YL and YL performed experiments and summarized the data; JL, YL and GG designed experiments; JL, LY, YL and GG wrote the paper; all authors have read and approved the final manuscript.

## Pre-publication history

The pre-publication history for this paper can be accessed here:

http://www.biomedcentral.com/1471-2407/12/370/prepub
